# An Egg Parasitoid Efficiently Exploits Cues From a Coevolved Host But Not Those From a Novel Host

**DOI:** 10.3389/fphys.2019.00746

**Published:** 2019-07-04

**Authors:** Valeria Bertoldi, Gabriele Rondoni, Jacques Brodeur, Eric Conti

**Affiliations:** ^1^ Dipartimento di Scienze Agrarie, Alimentari ed Ambientali, Università degli Studi di Perugia, Perugia, Italy; ^2^ Département de Sciences Biologiques, Institut de Recherche en Biologie Végétale, Université de Montréal, Montréal, QC, Canada

**Keywords:** *Trissolcus japonicus*, invasive species, *Halyomorpha halys*, herbivore-induced plant volatiles, predator, *Podisus maculiventris*, biological control

## Abstract

Egg parasitoids have evolved adaptations to exploit host-associated cues, especially oviposition-induced plant volatiles and odors of gravid females, when foraging for hosts. The entire host selection process is critical for successful parasitism and relevant in defining host specificity of parasitoids. We hypothesized that naïve egg parasitoid females reared on their coevolved host are able to exploit cues related to the coevolved host but not those from a novel host. We used the egg parasitoid *Trissolcus japonicus*, its coevolved host *Halyomorpha halys*, and the non-coevolved host *Podisus maculiventris* to evaluate this hypothesis. *H. halys*, a polyphagous pest native from Eastern Asia, has invaded North America and Europe, resulting in serious damage to crops. *T. japonicus* is the most effective egg parasitoid of *H. halys* in its native area and thus considered a major candidate for biological control. This parasitoid was detected in North America and Europe as a result of accidental introductions. Laboratory host range of *T. japonicus* includes *P. maculiventris,* an American predatory stink bug used as a biological control agent of several pests. Using *T. japonicus* reared on its natural host *H. halys*, we tested in a Y-tube olfactometer the responses of naïve parasitoid females to volatiles from tomato plants with a deposited egg mass and feeding punctures of either *H. halys* or *P. maculiventris*. Additionally, using two different olfactometer set-ups, we tested *T. japonicus* responses to volatiles emitted by eggs and mature males and females of *H. halys* or *P. maculiventris*. Tomato plants subjected to oviposition and feeding by *H. halys* were preferred by the wasp compared to clean plants, suggesting a possible activation of an indirect defense mechanism. Furthermore, *T. japonicus* females were attracted by cues from gravid females and mature males of *H. halys* but not from eggs. By contrast, naïve parasitoid females never responded to cues associated with *P. maculiventris*, although this non-target host is suitable for complete parasitoid development. Such lack of responses might reduce the probability of *T. japonicus* locating and parasitizing *P. maculiventris* under field conditions. Our experimental approach properly simulates the parasitoid host-location process and could be combined with the required host specificity tests for risk assessment in biological control programs.

## Introduction

Efficient exploitation of host-associated cues is a key feature for successful reproduction in insect parasitoids that need to invest their limited time on the location and parasitization of suitable hosts (Godfray, 1994; Vinson, 1998). This foraging process is especially challenging for egg parasitoids because their inactive and often inconspicuous hosts are hardly perceived from a distance (Vinson, 1998; Fatouros et al., 2008; Colazza et al., 2010). Egg parasitoid females have thus evolved adaptations to exploit host-related cues that are highly detectable and reliable indicators of the presence of suitable hosts, such as oviposition-induced plant volatiles and volatiles from gravid host females but also less reliable cues from males and nymphs (Hilker and Meiners, 2006; Conti and Colazza, 2012; Hilker and Fatouros, 2015).

In spite of such highly specialized adaptations, egg parasitoids often show a relatively wide host range, attacking species belonging to a given or related families, or even to different orders, on different plant species (Chantarasa-ard et al., 1984; Mansfield and Mills, 2002; Conti et al., 2004; Salerno et al., 2006; Zhang et al., 2011). Thus, foraging egg parasitoids can exploit different combinations of host-associated cues (Lewis et al., 1982; Vet and Groenewold, 1990; Turlings et al., 1993; Meiners and Hilker, 2000; Peñaflor et al., 2011). Alternatively, the nature of induced plant volatiles might be similar among different plant species, especially when plants belong to the same family (Colazza et al., 2004a,b; Mumm and Dicke, 2010).

Parasitoid host range may appear even wider under laboratory conditions, as some species show the capability to parasitize and develop within species that are not their natural hosts (Barratt et al., 1997; Babendreier et al., 2003). This aspect is interesting from an applied perspective since it allows rearing biocontrol agents on factitious hosts (Hoffmann et al., 2001; Cônsoli and Grenier, 2010) and consents the establishment of novel associations (Wyckhuys et al., 2009; Henry et al., 2010). On the other hand, this can be a limitation to classical biological control due to the apparently wide host range shown by these parasitoids in host-specificity tests, thus suggesting a risk that the candidate biocontrol agent may attack non-target species (Barron et al., 2003; Louda et al., 2003; Girod et al., 2018).

However, how high is the probability that, under field conditions, parasitoids would find and, subsequently, successfully parasitize non-target hosts? Considering that parasitoid host range is shaped not only by host recognition, acceptance, and suitability, but by the entire selection process, the role played by host-related cues in defining host specificity is relevant (Conti et al., 2004; Salerno et al., 2006). Exotic host species provide suitable models to examine the role of host selection cues in parasitoid host specificity. When an exotic herbivorous insect enters a new environment, multiple novel interactions may be established with native species, involving different trophic levels and resulting in unpredictable ecological consequences. From one side, native parasitoids (and other natural enemies) may adapt to the novel host, whereas from the other side, the invading species might disrupt the native trophic systems with possible negative consequences on natural control of native pest populations (Fand et al., 2013; Harvey, 2015; Martorana et al., 2017).

A relevant example is that of the brown marmorated stink bug (BMSB), *Halyomorpha halys* Stål (Hemiptera: Pentatomidae). Native to Eastern Asia, *H. halys* was first recorded in North America in 2003 (Hoebeke and Carter, 2003) and in Europe in 2004 (Wermelinger et al., 2008; Arnold, 2009; Haye et al., 2014). Since then *H. halys* has spread over the two continents and, because of its high polyphagy and capacity to build up large populations, it is causing serious damage to many agricultural systems (Hoebeke and Carter, 2003; Lee et al., 2013). In both invaded continents, several indigenous parasitoids and predators exploit the invasive herbivore, although their efficacy is generally low (Rice et al., 2014; Abram et al., 2015; Haye et al., 2015; Cornelius et al., 2016; Herlihy et al., 2016; Roversi et al., 2017; Costi et al., 2018; Morrison et al., 2018; Stahl, 2018). Interestingly, two native European parasitoids were found to be attracted to plant volatiles induced by oviposition of *H. halys* and to volatiles from males, which might be related to the low host specificity of these parasitoids (Rondoni et al., 2017). However, it has been documented both in America and Europe that *H. halys* may also act as an “evolutionary trap” for some native parasitoid species, which readily accept stink bug eggs as host but cannot successfully develop (Abram et al., 2014; Haye et al., 2015; Konopka et al., 2018). Additionally, *H. halys* may disrupt semiochemical networks, thus affecting the efficacy of local parasitoids (Martorana et al., 2017).

Therefore, in addition to relying on new associations with native parasitoids for control of *H. halys*, the introduction of coevolved parasitoids from the native area of the pest, in Asia, might be also considered. *Trissolcus japonicus* (Ashmead) (Hymenoptera: Scelionidae) is the dominant egg parasitoid of *H. halys* in its native area, with high parasitism rates (60–90%), and a candidate for biological control (Qiu et al., 2007; Yang et al., 2009; Dieckhoff et al., 2017). Nevertheless, its relatively large host range in Asia, together with results from host specificity tests indicating its ability to attack several *Pentatomoidea* species, are of concern to biocontrol practitioners (Haye et al., 2014; Lara et al., 2016; Matsuo et al., 2016; Hedstrom et al., 2017; Botch and Delfosse, 2018). Noticeably, the host range of *T. japonicus* in North America also includes *Podisus maculiventris* (Say) (Hemiptera: Pentatomidae) (Hedstrom et al., 2017; Botch and Delfosse, 2018), a predator of several insect pests including *H. halys* (Pote and Nielsen, 2017). When reared on *H. halys* eggs, *T. japonicus* showed a strong preference for its associated host (Botch and Delfosse, 2018). However, when reared on non-target hosts, this parasitoid showed reduced host-specificity, although a trade-off was observed in terms of reduced brood size and fertility, suggesting specialization to the coevolved host (Botch and Delfosse, 2018).

Because of such risks of non-target effects, *T. japonicus* has not yet been released in the field for biological control. In spite of that, this parasitoid is now present both in North America (Talamas et al., 2015; Milnes et al., 2016; Hedstrom et al., 2017; Morrison et al., 2018; Abram et al., 2019) and Europe (Stahl et al., 2018), likely as a consequence of accidental introductions (Milnes et al., 2016).

Therefore, it would be important to, first, confirm that *T. japonicus* exploits reliable volatile cues associated with its natural host *H. halys* and, second, determine if the parasitoid can also exploit cues associated with non-target *P. maculiventris*. It cannot be ruled out that the parasitoid could respond to cues from non-coevolved hosts as a consequence of learning (Giunti et al., 2015) or conditioning by rearing host (Godfray, 2007; Botch and Delfosse, 2018). However, our investigation is focused on naïve parasitoids reared on their naturally associated host *H. halys*. This species reliably represents the most available host for *T. japonicus* under field conditions in the invaded areas, where the population density of *H. halys* might be higher compared to that of the predatory stink bug *P. maculiventris.*

Here, we carried out olfactometer bioassays to test the following hypotheses: (1) naïve *T. japonicus* females have the innate capacity to efficiently exploit *H. halys* kairomones and oviposition-induced plant synomones and (2) although *T. japonicus* was shown to accept and develop in *P. maculiventris* eggs in the laboratory, naïve females reared on *H. halys* do not respond to cues associated with this new host because of lack of coevolution and learning experience. Using two types of Y-tube olfactometer set-ups (long- and close-distance assays), we tested the parasitoid response to plant and host volatiles, associated with *H. halys* and *P. maculiventris.*

## Materials and Methods

### Insect Rearing

Adults of *H. halys* were originally collected from a population in Hamilton (Ontario, Canada) in 2012 and reared continuously thereafter in nylon ventilated cages (30 cm^3^) with raw pumpkin seeds, carrots, green beans, grapes, and potted soybean plants, at 24 ± 1°C, 50 ± 5% relative humidity and a 16:8 h light:dark photoperiod. Eggs of *H. halys* were collected every 2 days and transferred to new cages to maintain the colony or used for parasitoid rearing. Eggs laid on the sides of the cages were collected daily for experiments.

Adults of *P. maculiventris* were originally collected from several locations in London and Ottawa (Ontario, Canada) regions in 2011 and 2012 and partly pooled with individuals provided from a greenhouse supply company (Anatis Bioprotection, Canada). Adults were fed with live mealworm larvae, *Tenebrio molitor* L. (Coleoptera: Tenebrionidae), fresh green beans, and bean plants, and reared in nylon cages at 24 ± 1°C, 50 ± 5% relative humidity and a 16:8 h light:dark photoperiod. Previous studies showed that *P. maculiventris* commonly feeds on plant tissues, especially when prey are scarce, probably to acquire water and nutrients (Ruberson et al., 1986; Valicente and O’Neil, 1995; De Clercq, 2008). First instar larvae, which do not feed, were provided with green beans for moisture. From the second instar onward, nymphs were kept in plastic cylinders and fed with mealworm and green beans. Eggs were collected every 2 days to maintain the colony and daily, from the sides of the cages, for experiments.

*T. japonicus* was obtained from the Beneficial Insects Introduction Research Unit, USDA Agricultural Research Service, Newark, DE, USA in 2017. Parasitoids were kept in nylon cages (30 cm^3^) and provided with a 1:1 (vol/vol) honey water solution on a small piece of ParaFilm®. *T. japonicus* was reared on 24 h-old *H. halys* eggs. Each week, egg masses were glued (Pritt® stick glue) on filter paper, offered to parasitoids, and then transferred to Petri dishes for parasitoid development. After emergence, male and female parasitoids were kept together for mating. For the experiments, insects were isolated in glass tubes before being tested.

### Plants

Seeds of tomato (*Lycopersicum esculentum* Mill. cv Rio Grande) were individually sown in plastic pots (Ø: 5.5–8 cm; h: 6.5 cm) filled with a mix of coarse sphagnum peat moss and perlite (Berger BM 6 soil). Plants were maintained in a climatic chamber (24 ± 2°C, 70 ± 5% RH, 12 h:12 h L/D) and irrigated every 3 days. A soluble mixture fertilizer (PlantProd 15N-30P-15K) was added 1 week after germination. Plants 3–4 weeks old were used for the olfactometer assays.

### Plant Volatiles

Tomato plants used as a source of volatiles in bioassays were exposed to a reproductive stink bug female of either *H. halys* or *P. maculiventris*. The pentatomid female was placed in a clip cage for 24–72 h and allowed to feed and oviposit on the plant. Plants were regularly checked for finding egg masses, and the female was removed after egg detection on the abaxial leaf surface, whereas eggs were not removed to prevent mechanical damage to plant (Colazza et al., 2004a,b; Fatouros et al., 2005; Conti et al., 2010). When eggs were not found after 72 h, the plant was considered as having being exposed to only feeding activity. To reduce feeding damage by *H. halys* on the plants and to promote oviposition, five sunflower kernels were placed in the clip cage. No prey was provided to *P. maculiventris* to ensure plant feeding by this predaceous stink bug. The plants were tested 24 h after oviposition or after the end of the exposition period (72 h) when there was only feeding. The four treatments were: P + HhFeed: plants with *H. halys* feeding damage but no oviposition; P + HhEggs: plants with *H. halys* feeding damage and oviposition of an egg mass; P + PmFeed: plants where a *P. maculiventris* female had fed but did not oviposit; P + PmEggs: plants where a *P. maculiventris* female had fed and laid an egg mass. Clean tomato plants (CP) were used as control.

### Stink Bug Volatiles

Volatile cues from males or females in their reproductive phase or freshly laid eggs of *H. halys* or *P. maculiventris* were tested in the olfactometer vs. clean air. Stink bug males were 1–2 weeks old, sexual maturity occurring about 1 week following emergence (Polajnar et al., 2016). Females were at least 1-week old and in their ovipositional phase, as evident by their physogastric abdomen (De Clercq, 2008; Polajnar et al., 2016). Four adult pentatomids (females or males) were used per bioassay, and adults were not fed during the assays. Tested egg masses of either *H. halys* or *P. maculiventris* were <24 h old and composed of about 28 and 19 eggs, respectively, as naturally laid on the nylon sides of the rearing cages. The six treatments were volatiles from: *Hh*Fem: four *H. halys* females; *Hh*Mal: four *H. halys* males; *Pm*Fem: four *P. maculiventris* females; *Pm*Mal: four *P. maculiventris* males; *HhEggs*: a single egg mass of *H. halys*; and *PmEggs*: a single egg mass of *P. maculiventris*. Clean air was used in the control arm of the olfactometer against insect volatiles.

### Olfactometer Bioassays

A Y-tube olfactometer, made from a glass body (stem: 80 mm; arms: 200 mm at 30° angle between arms; internal diameter 12 mm; outside diameter 15 mm), was used to determine the behavioral responses of female *T. japonicus* to host-associated cues. A stream of air from the laboratory compressed-air supply was purified by a charcoal filter made from a 500 ml flask (Pyrex) with four layers each of alternated charcoal and fiberglass. The stream was then bubbled through water in a second 500 ml flask (Pyrex) to humidify the air before it passed into the olfactometer. The air also passed through flow meters set in each arm at 0.5/0.6 L min^−1^. The Y-tube was held in a cardboard box, black on the sides and white on bottom, with two holes for connection with the air flow tubes, and illuminated from above by two 18-W cool white fluorescent lamps. A thin pencil line drawn on the base of the olfactometer box at 100 mm from the beginning of stem divided the olfactometer into three parts: the left arm, the right arm, and the common area with the junction of the two arms.

Two different types of olfactometer setups were used: “long-distance” and “close-distance” olfactometer. In the “long-distance” setup, each air stream passed through a 4 L glass jar (Ø: 10–15 cm; h: 30 cm) containing the odor source and connected to the olfactometer arm through a plastic tube (inner diameter 8 mm and outer diameter 12 mm) of about 40 cm of length. For the “close-distance” setup, the source of volatiles were placed close to the olfactometer in two little chambers, each made from a modified 15 ml falcon tube with two holes, one on the cover and one on the bottom, both closed by a brass mesh. These chambers were connected with the tubes carrying the air and placed directly at the ends of the arms of the olfactometer, held with Parafilm wrapped on it. To avoid visual stimuli the chambers were kept outside from the illuminated box containing the olfactometer and in the dark. This type of set-up, similar to others used by Colazza et al. (1999), Conti et al. (2003) and Zhong et al. (2017), aimed at studying the searching behavior of the parasitoid once arrived close to the host colony. The volatiles from stink bug treated plants were tested in the “long-distance” olfactometer, whereas those from adults and eggs of *H. halys* and *P. maculiventris* were tested in both olfactometers. The rationale being that oviposition-induced plant volatiles are probably exploited from a longer range than volatiles from adult stink bugs and from eggs (Conti et al., 2003; Colazza et al., 2010; Hilker and Fatouros, 2015).

The assays were conducted from the 9:00 to 16:00, and the temperature in the bioassay room was maintained at 25°C. A *T. japonicus* female was introduced in the Y-tube at the entrance of the central stem and let move freely for 10 min. After up to five wasps (block) had been tested, the glass olfactometer was cleaned with a laboratory detergent and rinsed with hot tap water. Moreover, the tubes connecting the plants to each of the Y-tube arms were switched to avoid bias. All tested wasps were naïve, 3–8 days old, likely mated, and used only once. Each plant, group of adults or egg masses were used for a set of up to 10 bioassays, each carried out with different parasitoid females. For each treatment, 29–77 replicates were conducted. Overall, only five insects did not respond, i.e., they stayed in the common stem, and were removed from the analysis. The parasitoid’s residence time, i.e., the durations and frequency of their presence in each olfactometer arm and in the common stem, was recorded using JWatcher 1.0 (Blumstein et al., 2006).

### Data Analyses

For each treatment, percent residence time was calculated as the proportion of time spent in the treatment arm on the total time spent in both arms (i.e., excluding the time spent in the common stem). Percent data were normalized using arcsine square root transformation. Generalized linear models (GLMs, Gaussian error distribution) were fitted to test the attraction of *T. japonicus* females to the different sources of volatiles against clean air or clean plant (Zuur et al., 2009). The effect of blocks was initially included as a random effect in generalized mixed models, but its relevance, evaluated by means of likelihood ratio test, was never justified (Zuur et al., 2009). All analyses were run under R statistical environment (R Core Team, 2014).

## Results

### “Long-Distance” Assays

**Figure 1:**
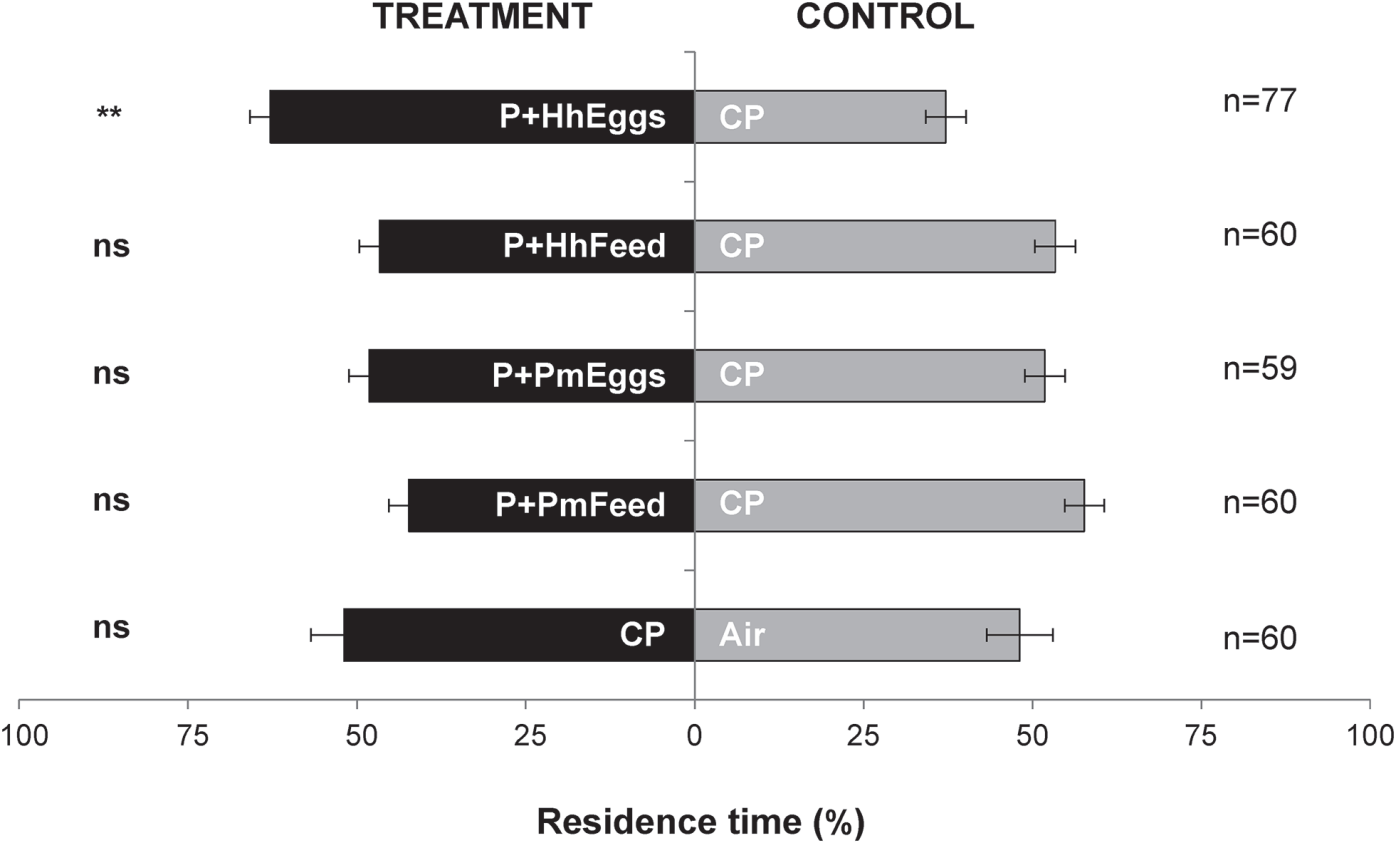
Residence time (mean percentage ± SE) of *Trissolcus japonicus* females in the treatment vs. control arm of a Y-tube olfactometer designed for “long-distance” assays. Treatments were volatiles from tomato plants, where: a *Halyomorpha halys* female had fed and oviposited (P+HhEggs); a *H. halys* female had fed but did not oviposit (P+HhFeed); a *Podisus maculiventris* female had fed and oviposited (P+PmEggs); a *P. maculiventris* female had fed but did not oviposit (P+PmFeed). Volatiles from clean tomato plants were used as controls (CP). Data were analyzed by means of GLMs (^**^P < 0.01; ns: not significant).

Females *T. japonicus* responded to volatiles from tomato plants with feeding damage and an egg mass of *H. halys* (P + HhEggs vs. CP: *p* = 0.0013) but not to volatiles from plants with only feeding punctures by *H. halys* (P + HhFeed vs. CP: *p* = 0.382) (Figure 1). In contrast, parasitoid females did not respond to volatiles from plants with feeding damage and an egg mass of *P. maculiventris* (P + PmEggs vs. CP: *p* = 0.522). However, they seemed to be repelled by plants subjected only to feeding by *P. maculiventris* although the results were barely non-significant (P + PmFeed vs. CP: *p* = 0.0567). Finally, the parasitoids did not show a preference for clean control plants or clean air (CP vs. Air: *p* = 0.659) (Figure 1).

**Figure 2:**
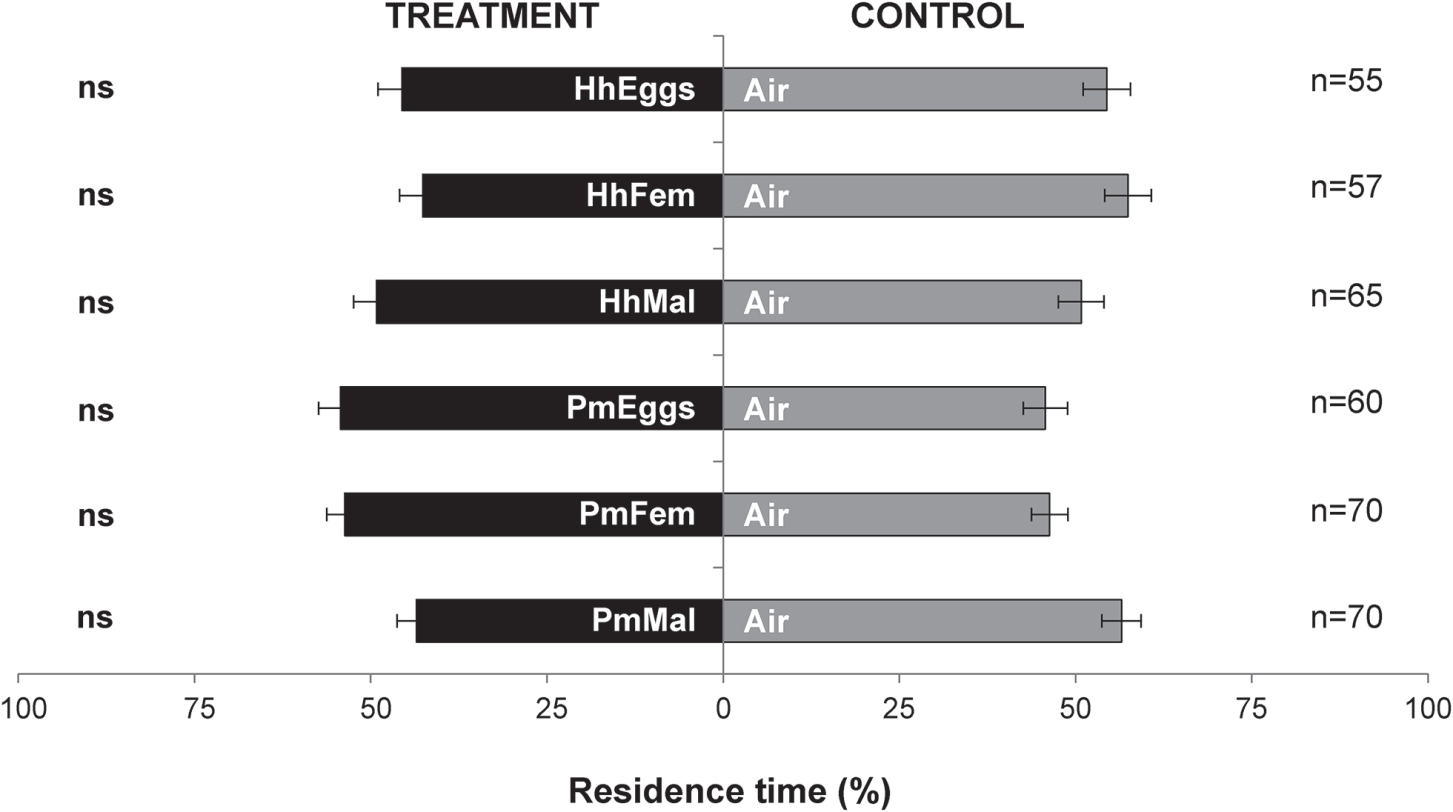
Residence time (mean percentage ± SE) of *Trissolcus japonicus* females in the treatment vs. control arm of a Y-tube olfactometer designed for “long-distance” assays. Treatments were volatiles of: a single egg mass of *Halyomorpha halys* (HhEggs); *H. halys* gravid females (HhFem); *H. halys* mature males (HhMal); a single egg mass of *Podisus maculiventris* (PmEggs); *P. maculiventris* gravid females (PmFem); *P. maculiventris* mated males (PmMal). Air was used as control (Air). Data were analyzed by means GLMs (ns: not significant).

Parasitoid females did not respond in the “long-distance” olfactometer setup to volatiles emitted by *H. halys* eggs (HhEggs vs. Air: *p* = 0.258), gravid females (HhFem vs. Air: *p* = 0.119) or reproductive males (HhMal vs. Air: *p* = 0.848). Similarly, the parasitoid was not attracted to volatiles from *P. maculiventris* (PmEggs vs. Air: *p* = 0.407; PmFem vs. Air: *p* = 0.297; PmMal vs. Air: *p* = 0.090) (Figure 2).

### “Close-Distance” Assays

**Figure 3:**
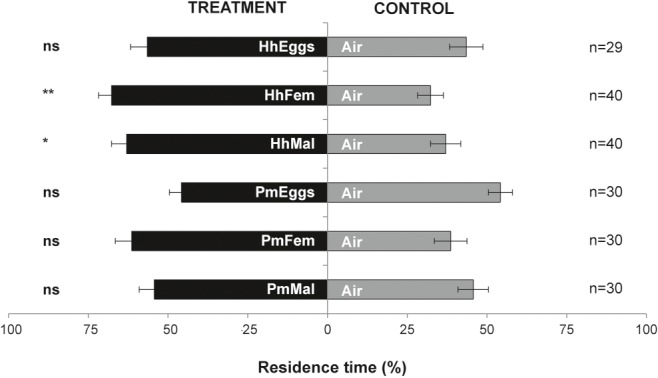
Residence time (mean percentage ± SE) of *Trissolcus japonicus* females in the treatment vs. control arm of a Y-tube olfactometer designed for “close-distance” assays. Treatments were volatiles of: a single egg mass of *Halyomorpha halys* (HhEggs); *H. halys* gravid females (HhFem); *H. halys* males (HhMal); a single egg mass of *Podisus maculiventris* (PmEggs); *P. maculiventris* gravid females (PmFem); *P. maculiventris* males (PmMal). Air was used as control (Air). Data were analyzed by means of GLMs (^**^p < 0.01; ^*^p < 0.05; ns: not significant).

Females of *T. japonicus* were not attracted in the “close-distance” olfactometer setup to volatiles from *H. halys* eggs (HhEggs vs. Air: *p* = 0.356). In contrast, they significantly responded to cues from *H. halys* gravid females (HhFem vs. Air: *p* = 0.0052) and mature males (HhMal vs. Air: *p* = 0.0487). However, *T. japonicus* females never responded to *P. maculiventris* in the “close-distance” olfactometer setup (PmEggs vs. Air: *p* = 0.439; PmFem vs. Air: *p* = 0.129; PmMal vs. Air: *p* = 0.620) (Figure 3).

## Discussion

Here, we demonstrated using “long-distance” olfactometer assays that naïve females of the egg parasitoid *T. japonicus*, reared from its coevolved host *H. halys*, positively respond to volatiles emitted by tomato plants being fed upon and bearing an egg mass of *H. halys*. Using “close-distance” olfactometer assays, we showed that the parasitoids have the capacity to detect and positively respond to cues associated with gravid females and mature males of *H. halys*. In contrast, naïve *T. japonicus* females reared on *H. halys* did not respond to any of the eight treatments involving odors associated with *P. maculiventris*. These results confirm our hypothesis about the capacity of the naïve egg parasitoid, reared on *H. halys*, to exploit cues related to its coevolved host but not those related to the novel association. However, we cannot exclude that experienced parasitoid females or those reared on *P. maculiventris* may also respond to cues from this novel host (Tognon et al., 2014, 2018; Botch and Delfosse, 2018). Additionally, in the “close-distance” setup, the parasitoid showed an apparently similar response to *P. maculiventris* females and *H. halys* males; thus we cannot exclude that a higher number of replications in the former would have resulted in significant values.

The ability of *T. japonicus* to exploit plant volatiles associated with *H. halys* eggs may be consistent with previous findings on host selection strategies developed by egg parasitoids in general and, more specifically, those attacking pentatomids (reviewed by Conti and Colazza, 2012), although we cannot exclude parasitoid response to a combination of plant and egg volatiles rather than just plant volatiles. An “early herbivore alert” (*sensu* Hilker and Meiners, 2006) by the plant denotes a particular type of indirect induced defense that has been observed in several other systems (Colazza et al., 2004a; Conti et al., 2010; Peñaflor et al., 2011). In our experiments, tomato plants subjected to *H. halys* oviposition and feeding were preferred over clean plants by females of *T. japonicus* in olfactometer assays, whereas those subjected only to feeding were not. Similarly, in a different system, *Trissolcus basalis* (Wollaston) responded to leguminous plants on which *N. viridula* had fed and oviposited compared to control plants, but not to plants on which the host only had fed (Colazza et al., 2004a). The emission of volatiles from leguminous plants was systemic, thus maximizing the release surface and synomone volatilization; additionally, the synomone activity was tuned to parasitoid behavior since preference was higher toward fresh eggs and faded for older eggs that were not suitable for parasitoid development (Colazza et al., 2004a). Chemical analyses of the emitted volatiles showed an increase of (E)-β-caryophyllene (Colazza et al., 2004b). However, of significance, tomato plants that have been used in our system are not native from Asia, although their cultivation has spread worldwide from America in the 16th century, including Eastern China. Thus, tomato defensive responses to *H. halys* may be quite general, against several species, or might result from plant adaptation, although further investigations may provide different explanations.

Interestingly, previous papers showed responses of different parasitoids to volatiles emitted by the plant-herbivore complex and the activation of plant defenses against *H. halys* (Rondoni et al., 2017, 2018). Specifically, the egg parasitoids *Anastatus bifasciatus* (Geoffroy) (Hymenoptera: Eupelmidae) and *Ooencyrtus telenomicida* (Vassiliev) (Hymenoptera: Encyrtidae) positively responded in an olfactometer to volatiles emitted by faba bean plants with oviposition and feeding punctures of *H. halys* and to volatiles from males (Rondoni et al., 2017). Considering that *T. basalis* did not react to *H. halys* induced plant volatiles, exploitation of plant volatiles in this system most likely depends on parasitoid host range, which is much wider for *A. bifasciatus* and *O. telenomicida* compared to *T. basalis* (Rondoni et al., 2017). Thus, complex combinations of indirect (Rondoni et al., 2017) and direct (Rondoni et al., 2018) defense strategies can be activated by plants against invasive herbivore species, suggesting possible existence of general (non-specific), conserved plant defensive mechanisms. One of our olfactometer set-ups for the plant volatile assays tentatively simulated a long-range diffusion of the chemical blend from plants. *T. japonicus* response seems thus consistent with general hypothesis that induced-plant volatiles act as long-distance attractants (Vet and Dicke, 1992; Hilker and Fatouros, 2015), although this is not always the case since in different systems female egg parasitoids only respond to oviposition-induced plant volatiles from a very short distance in static olfactometer (Fatouros et al., 2005, 2009; Conti et al., 2010). Therefore, additive effects from different cues, originated from host and plant, are worth being investigated for *T. japonicus* on plants attacked by *H. halys*.

In our experiments, *T. japonicus* also responded to cues from gravid females and sexually mature males of *H. halys*, but only when using the “close-distance” olfactometer set up, with the volatile sources at the end of the olfactometer arm, whereas it did not respond to host odors in the “long-distance” setup. The reason for these different responses is not clear and possibly depends on the different distance of source from the olfactometer, different concentrations of chemical volatiles or emissions of volatile blends based on the different container sizes and insect crowding conditions, or on a combination of these and other factors. The chemical ecology of *H. halys* has been intensely studied and several compounds have been identified (Khrimian et al., 2014; Harris et al., 2015; Weber et al., 2017; Nixon et al., 2018). Zhong et al. (2017) found that females of *T. japonicus* were attracted in Y-tube assays by n-tridecane, a component of the *H. halys* defensive secretion, and that treatment with this compound significantly improved host-searching efficiency of female *T. japonicus*. By contrast, (E)-2-decenal, also a defensive secretion, was strongly repellent to the parasitoid (Zhong et al., 2017). Therefore, additional investigation will be necessary to understand the role of host-derived cues acting on a short range in host location by *T. japonicus*, including possible vibrational cues from *H. halys* adults, as was shown for other egg parasitoids associated with stink bugs (Laumann et al., 2007, 2011).

Detection of host eggs by parasitoids is more difficult than for larval or adult hosts, as eggs emit small amounts of volatiles, mostly useful as short-range cues (Vet et al., 1995; Vinson, 1998). Accordingly, we did not observe any preference for volatiles from a single egg mass of *H. halys* in the bioassays. This also suggests that *T. japonicus* response to plants with *H. halys* eggs was due to the volatiles emitted from plants as a consequence of oviposition, although it cannot be excluded that the parasitoid is attracted by a combination of both plant and egg volatiles. However, while visual cues may have a role when the parasitoid is close to the egg mass (Sugimoto et al., 1988), they do not appear crucial for egg location, whereas very short-range volatile kairomones from eggs are considered more important, as shown for the egg parasitoid *T. brochymenae* on *M. histrionica* (Conti et al., 2003).

The lack of response by *T. japonicus* to volatiles from any treatments involving *P. maculiventris*, although it was expected from a coevolutionary and behavioral/learning perspective, still needs to be explained from a semiochemical perspective. *P. maculiventris* is a predatory stink bug that also feeds on plant tissues (Ruberson et al., 1986; Valicente and O’Neil, 1995), but no evidence of feeding damage is reported (De Clercq, 2008). In laboratory conditions, *P. maculiventris* often landed on tomato plants to lay eggs and feed (Bertoldi, personal observations). Intriguingly, in our olfactometer observations, tomato plants subjected to feeding by *P. maculiventris* but without oviposition seemed to be almost repellent toward *T. japonicus.* One may consider that plants have the ability to discriminate between herbivorous and predaceous stink bugs and thus emit synomones only when attacked by herbivores, but no data support this speculation. In any case, it seems that oviposition and feeding activity of *P. maculiventris* did not activate the plant indirect defenses or that the parasitoid is not attracted to the combination of volatiles from plant and *P. maculiventris* eggs.

Concerning treatments with only volatiles associated with reproductive females or males of *P. maculiventris*, naïve *T. japonicus* females reared from *H. halys* eggs did not respond in either types of olfactometer. This absence of innate response is not surprising, because of the lack of coevolution of the novel host-parasitoid association, and may be explained by the *P. maculiventris* volatiles, which are mostly different from those of *H. halys* (Aldrich et al., 1984; Kitamura et al., 1984; Harris et al., 2015; Zhong et al., 2017).

From an applied perspective, our results are interesting in the context of *T. japonicus* being the most effective parasitoid of *H. halys* in its native area (Yang et al., 2009; Dieckhoff et al., 2017), as well as a candidate for classical biological control against *H. halys* in newly invaded areas. This parasitoid shows a number of positive attributes, such as high parasitism rate in the field (Qiu et al., 2007; Talamas et al., 2013), short developmental time (Yang et al., 2009), cold tolerance (Santacruz et al., 2017), and climate suitability (Avila and Charles, 2018).

In spite of the concerns raised by the large host range of *T. japonicus*, which involves several stink bug species including *P. maculiventris* (Haye et al., 2014; Matsuo et al., 2016; Hedstrom et al., 2017; Botch and Delfosse, 2018), our results suggest that the probability for *P. maculiventris* to be located by the exotic parasitoid in the field would be low; *T. japonicus* will mainly respond to volatile cues from *H. halys*. This should limit parasitism risk to *P. maculiventris* and potentially to other native Pentatomid species, although it might be possible for *T. japonicus* to detect and parasitize *P. maculiventris* especially where *H. halys* co-exist with other *Pentatomidae*. Additionally, *T. japonicus* was shown to adapt to new hosts or learn to respond to its odor and after that become more prone to choose the new host, reducing its specificity, although there is a cost for such adaptation (Botch and Delfosse, 2018). The effect of the rearing host on parasitoid behavior was also described for other species (Godfray, 2007; Tognon et al., 2014, 2018). Further studies are therefore required to evaluate the physiological and the ecological host range of *T. japonicus* in the areas of introduction and to evaluate the possible effects of adaptation to the new host on the parasitoid ability to exploit host associated volatiles. Testing parasitoid responses to host cues, whether they are newly introduced or candidate for released in biological control programs, would help to predict possible non-target effects. We think that this approach could be complementary to standard host-specificity tests because it examines different host selection steps resulting in a given host-parasitoid association.

## Author Contributions

VB, GR, JB, and EC conceived and designed the experiments. VB and GR performed the experiments and analyzed the data. VB, GR, JB, and EC interpreted results, drafted, and revised the article.

### Conflict of Interest Statement

The authors declare that the research was conducted in the absence of any commercial or financial relationships that could be construed as a potential conflict of interest.
